# Supplementary material for: The Aqueous Leaf Extract of the Medicinal Herb Costus speciosus Suppresses Influenza A H1N1 Viral Activity under In Vitro and In Vivo Conditions
Source: Viruses. 2023 Jun 15;15(6):1375. doi: 10.3390/v15061375 (PMC10303568; doi:10.3390/v15061375)
Supplement: Supplementary file 1 [file viruses-15-01375-s001.zip › viruses-2337358-supplementary.pdf]

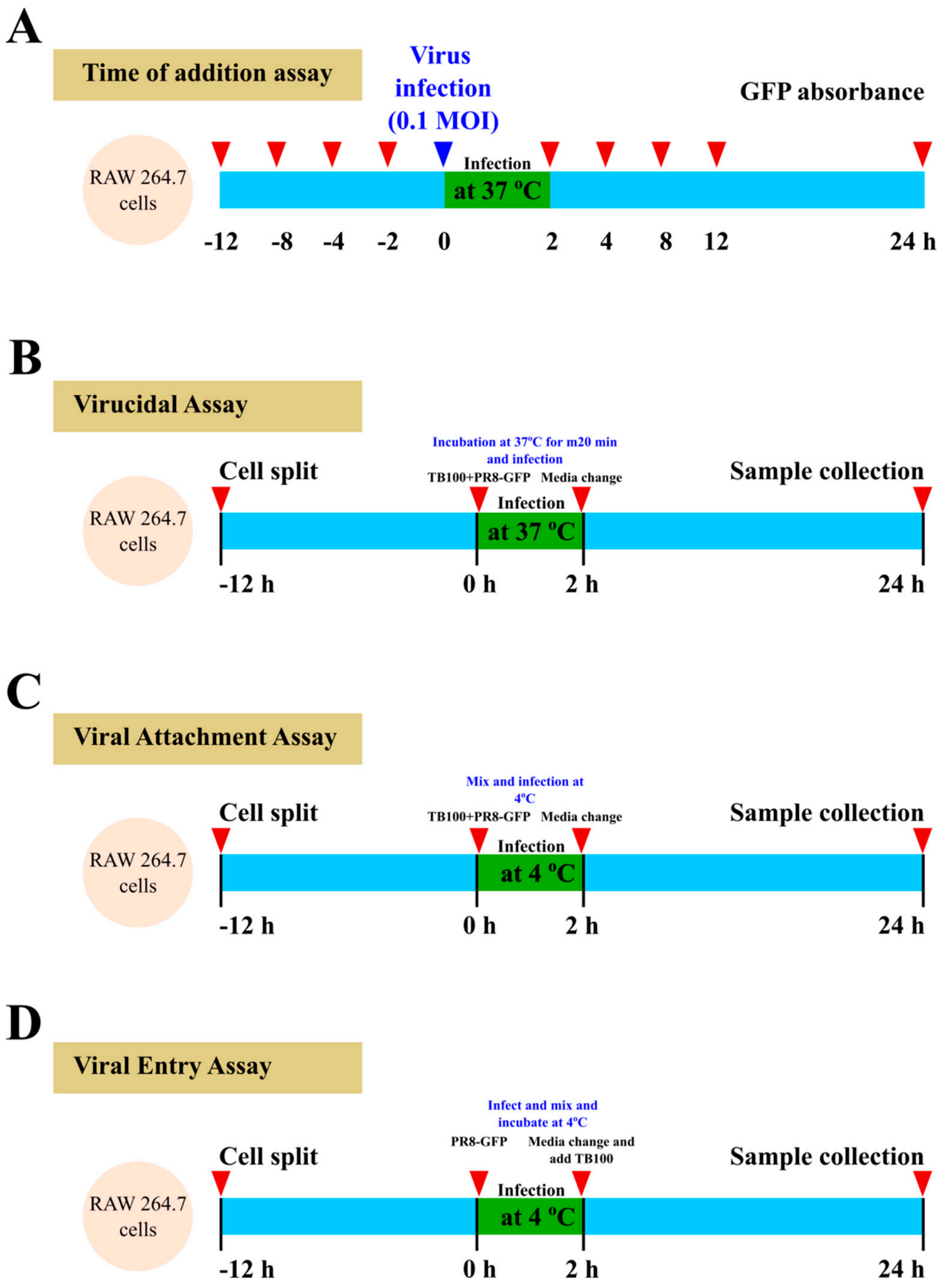

Figure S1. Treatment schedule used in TB100 time of addition and its effect on the influenza virucidal, viral attachment and entry.

Scheme

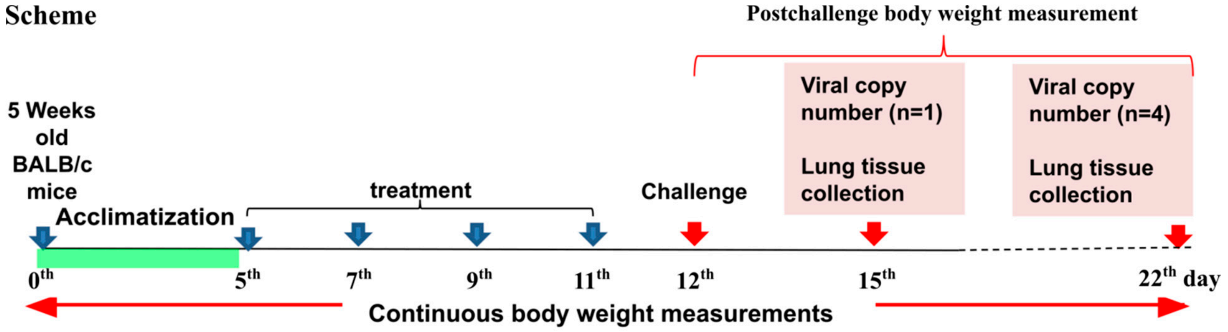

Group 1: TB100-1.05 mg/kg, 2: TB100-2.1 mg/kg, 3: TB100-4.2 mg/kg, 4: TB100-8.4 mg/kg, 5: Virus Only (VO), 6: Naive

Figure S2. TB100 inoculation schedule for BALB/c mice in vivo dose optimization study. Five weeks old female BALB/c mice were acclimatized for 5 days and TB100 was orally treat with TB100 variable concentrations. Mice were randomly divided for 6 groups (group 1: TB100-1.05 mg/kg, 2: TB100-2.1 mg/kg, 3: TB100-4.2 mg/kg, 4: TB100-8.4 mg/kg, 5: VO, 6: Naive). Four consecutive oral inoculations were conducted on 5th, 7th, 9th and 11th days. Nasal challenge with A H1N1 was conducted on 12th day. Lung tissue collection for histopathological analysis and TCID50 determination was done on the 15th and 22th day. Postchallenge body weight measurements were conducted on daily basis until the end.

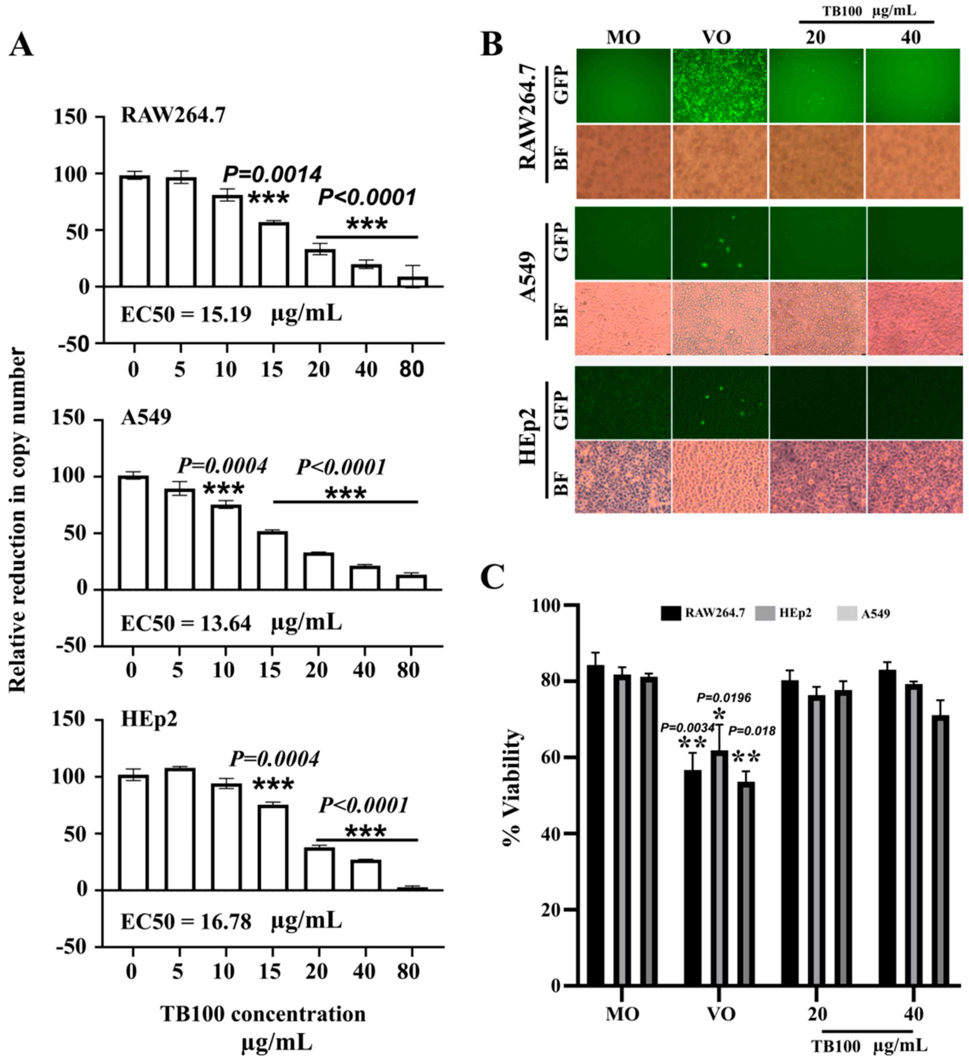

Figure S3. EC50 of TB100 on phagocytic and none phagocytic cells. (A) 50% effective concentration of TB100 was determined by viral copy number reduction. *** indicate significant difference against O h time point. (B) Representative fluorescence image showing the extent of infection observed in RAW264.7, A549 and HEp2. (C) TB100 effect on postinfected cell viability was evaluated using trypan blue exclusion method. Stars indicate significant difference against the TB100 non-treated control (qRT-PCR) or MO control (viability assay). Level of significance was determined at p<0.05. *, ** indicate significant difference against the MO control. Experiment was conducted in three independent trials. Mean ± SD is demonstrated.

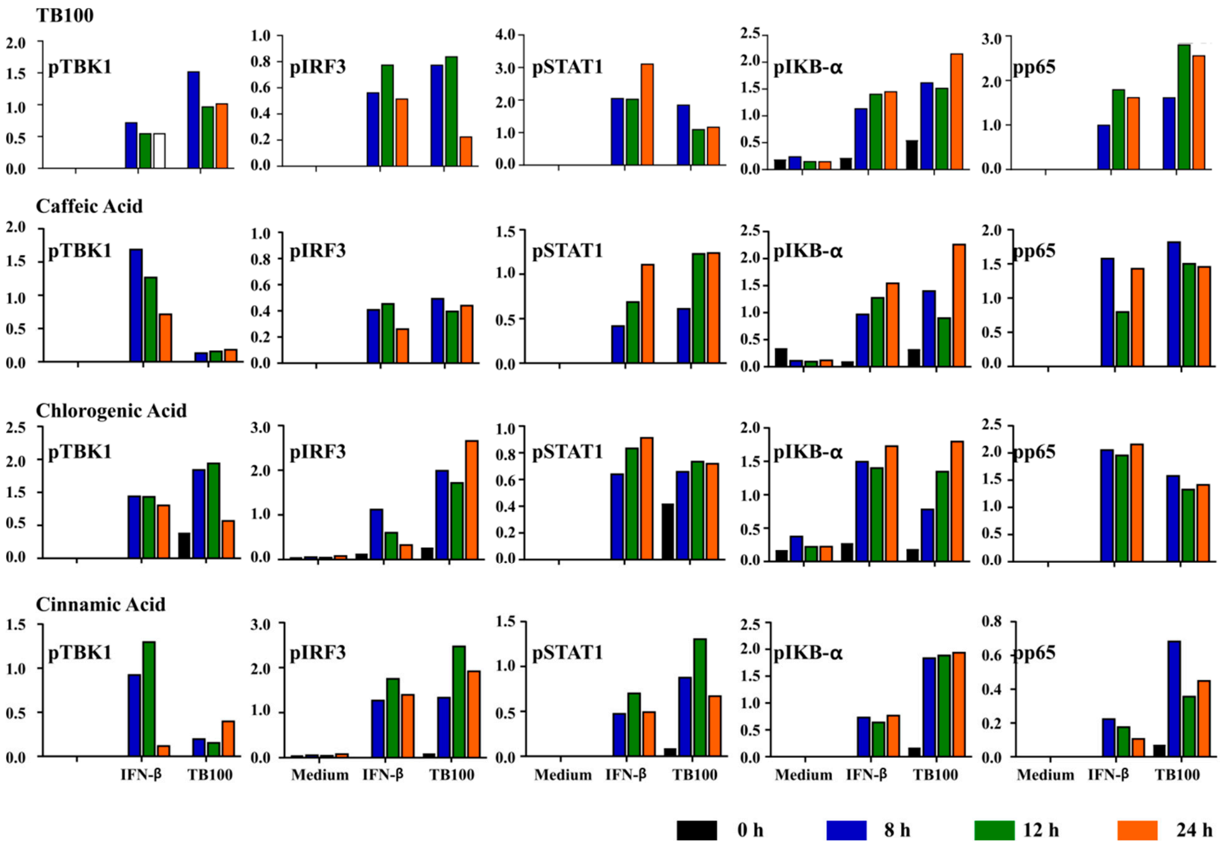

Figure S4. Quantification of western blot data. Image J software was utilized to quantify the band intensity. The results were normalized against the media alone control.

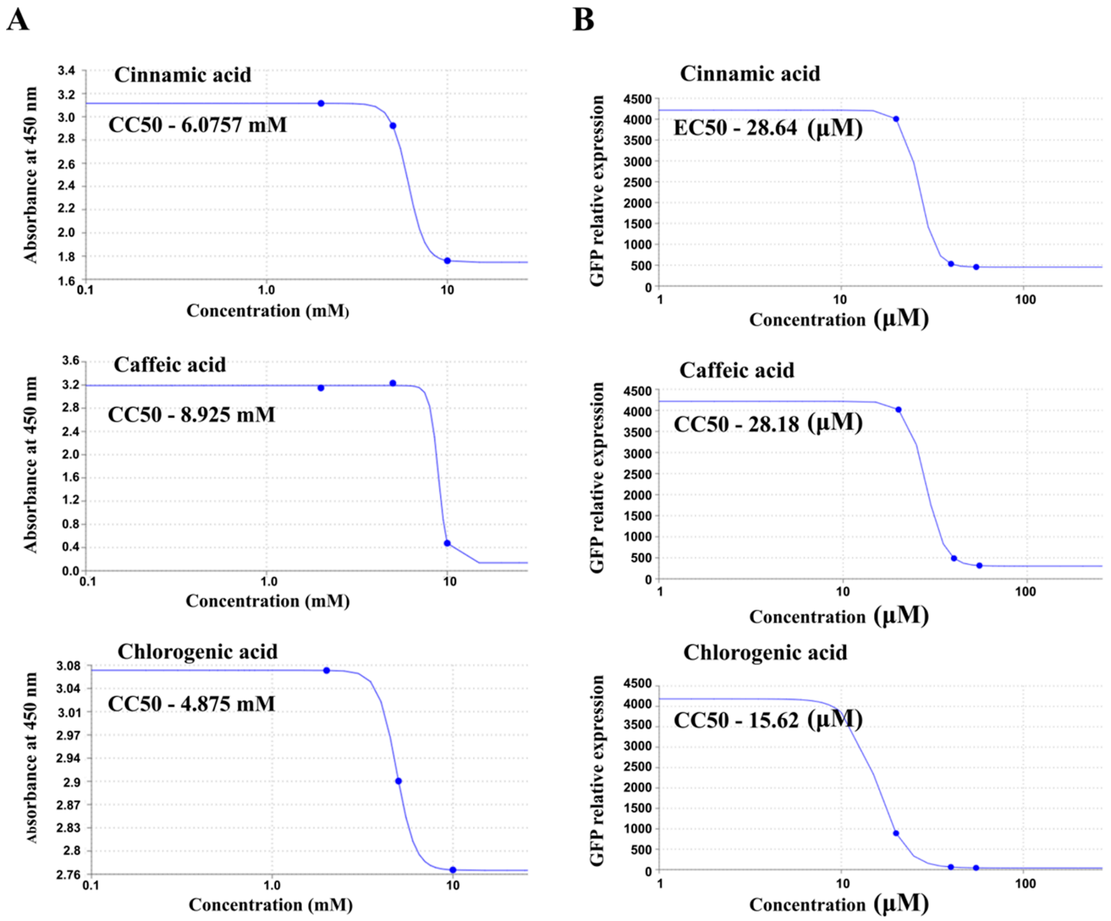

Figure S5. Determination of CC50 and EC50 of active compound identified in TB100 (A) 50% cytotoxic concentration of cinnamic acid, caffeic acid, and chlorogenic acid was determined by Ez-Cytox cytotoxicity assay. (B) 50% Effective concentration of active compounds was determined by GFP fluorescence reduction assay in RAW264.7 cells after in vitro infection with influenza A H1N1 PR8-GFP.

Table S1. Inoculation scheme for in vivo dose optimization study of TB100

Experimental Group	Animal Model	Animals per Group	Inoculation Dose/mice	Route	Challenge (Nasal Route)
TB100-0.1 mg/mL	BALB/c, female	5	0.02 mg (in 200 μL PBS)	Oral	2 × LD50
TB100-0.2 mg/mL	″	5	0.04 mg (in 200 μL PBS)	Oral	2 × LD50
TB100-0.4 mg/mL	″	5	0.08 mg (in 200 μL PBS)	Oral	2 × LD50
TB100-0.8 mg/mL	″	5	0.16 mg (in 200 μL PBS)	Oral	2 × LD50
PBS	″	5	200 μL PBS	Oral	2 × LD50
Naive	″	5	-	-	-

Table S2. Inoculation scheme for in vivo challenge study of TB100

Experimental Group	Animal Model	Animals per Group	Inoculation Dose/mice	Route	Challenge *(Nasal Route)
Naive	BALB/c, female	8	-	Oral	2 × LD50
PBS-H1N1	,,	8	200 μL PBS	Oral	2 × LD50
PC-H1N1	,,	8	1000 U of IFN-β	Nasal	2 × LD50
TB100-H1N1	,,	8	0.16 mg (in 200 μL PBS)	Oral	2 × LD50
PBS-H3N2	,,	8	200 μL PBS	Oral	2 × LD50
PC-H3N2	,,	8	1000 U of IFN-β	Nasal	2 × LD50
TB100-H3N2	,,	8	0.16 mg (in 200 μL PBS)	Oral	2 × LD50
PBS-H9N2	,,	8	200 μL PBS	Oral	2 × LD50
PC-H9N2	,,	8	1000 U of IFN-β	Nasal	2 × LD50
TB100-H9N2	,,	8	0.16 mg (in 200 μL PBS)	Oral	2 × LD50

*Note: Challenge was done using A H1N1, A H3N2 and A H9N2 influenza strains at 2 xLD50.
